# P-133. *Klebsiella pneumoniae* liver abscess as the herald of underlying colon neoplasm

**DOI:** 10.1093/ofid/ofae631.338

**Published:** 2025-01-29

**Authors:** Yi-Tsung Lin, Chien Chuang, Tsen-En Liao, Sheng-Hua Chou, Yu-Chien Ho, Szu-Yu Liu, Chih-Han Juan

**Affiliations:** Division of Infectious Diseases, Department of Medicine, Taipei Veterans General Hospital, Taipei, Taiwan, Taipei, Taipei, Taiwan (Republic of China); Taipei Veterans General Hospital and National Yang Ming Chiao Tung University, Taipei, Taipei, Taiwan; National Yang Ming Chiao Tung University, Taipei, Taiwan, Taipei, Taipei, Taiwan; Institute of Emergency and Critical Care Medicine, National Yang-Ming University, Taipei, Taiwan, Taipei, Taipei, Taiwan (Republic of China); Taipei Veterans General Hospital, Taipei, Taipei, Taiwan (Republic of China); Taipei Veterans General Hospital, Taipei, Taipei, Taiwan (Republic of China); Taipei Veterans General Hospital and National Yang Ming Chiao Tung University, Taipei, Taipei, Taiwan

## Abstract

**Background:**

*Klebsiella pneumoniae* liver abscess (KPLA) is an endemic disease in Eastern Asia, especially Taiwan. Hypervirulent *K. pneumoniae* strain in the gut is able to translocate across the colonic mucosal defects to the portal system, resulting in subsequent liver abscess. Occult colon cancer in patients with KPLA has been described in several case series. However, data regarding colonoscopic findings in patients with KPLA in Taiwan have not been investigated. We aimed to investigate whether the diagnosis of KPLA serves as a marker of underlying colorectal neoplasm.

**Methods:**

Consecutive patients with KPLA in Taipei Veterans General Hospital from April 2011 to January 2024 were included in this retrospective study. Clinical data were collected and colonscopy was recommended within 6 months of diagnosis of KPLA according to the patient’s will. Hypervirulent strains were defined as those with *rmpA* or *rmpA2*. In patients receiving colonoscopy, we determined the risk factors of underlying colon adenoma/adenocarcinoma using a logistic regression analysis.

**Results:**

A total of 491 unique KPLA episode was collected during the study period. Most of the available strains (401 of 422, 95%) were hypervirulent strains, and most of them (339 of 422) belonged to capsular type K1 or K2. One hundred and thirteen patients (23%) had undergone colonoscopy within six months of diagnosis of KPLA. Clinical characteristics were similar between patients who had undergone colonoscopy or not. The findings of colonoscopy showed that 48 patients had adenomas (42.5%) and 12 patients had adenocarcinoma (10.6%). Patients with colon adenoma/adenocarcinoma had a higher proportion of age over 50 years (90% versus 62.3%, P< 0.001) and female (45% versus 26.4%, P=0.04), compared with patients without colon adenoma/adenocarcinoma. The proportion of hypervirulent strains and the distribution of capsular types were similar between the 2 groups. In multivariate analysis, age over 50 years was the only independent risk factor for colon adenoma/adenocarcinoma (P=0.002, OR=5.14, 95% CI=1.84-14.36).

**Conclusion:**

Colon adenoma and adenocarcinoma were notably common in patients with KPLA. We recommended colonoscopy be routinely performed in patients with KPLA in the endemic area, especially for those older than 50 years old.

**Disclosures:**

**All Authors**: No reported disclosures

